# Fatty Acid Binding Protein 4 Could Be a Linking Biomarker Between Periodontitis and Systemic Diseases

**DOI:** 10.3390/biomedicines13020402

**Published:** 2025-02-07

**Authors:** Jiwon Song, Soo-Min Ok, Eun-Young Kwon, Hyun-Joo Kim, Ju-Youn Lee, Ji-Young Joo

**Affiliations:** 1 Department of Periodontology, Dental Research Institute, Pusan National University Dental Hospital, Yangsan 50612, Republic of Korea; songjiwone@naver.com (J.S.); periohjkim@pusan.ac.kr (H.-J.K.); heroine@pusan.ac.kr (J.-Y.L.); 2 Department of Oral Medicine, Dental Research Institute, Pusan National University Dental Hospital, Yangsan 50612, Republic of Korea; oksoomin@pusan.ac.kr; 3 Department of Periodontology, Dental Clinic Center, Pusan National University Hospital, Busan 49241, Republic of Korea; betteryoung@hanmail.net; 4 Department of Periodontology, Periodontal Disease Signaling Network Center, School of Dentistry, Pusan National University, Yangsan 50612, Republic of Korea

**Keywords:** atherosclerosis, diabetes mellitus, fatty acid binding protein, periodontal disease, periodontitis, type 2 diabetes

## Abstract

**Background/Objectives**: This study aims to investigate the relationship between serum fatty acid-binding protein 4 (FABP4) levels and the severity of periodontitis in systemically healthy individuals. Additionally, the study examines whether non-surgical periodontal treatment can reduce FABP4 levels, establishing its potential as a biomarker linking periodontitis to systemic diseases. **Methods**: A total of 89 participants with stage I, II, or III periodontitis were recruited, excluding individuals with systemic diseases. Clinical parameters such as clinical attachment level (CAL), probing depth (PD), and gingival index (GI) were recorded. Serum FABP4 levels and *Porphyromonas gingivalis* (*P. gingivalis*) antibody titers were measured before and after periodontal treatment using ELISA kits. Statistical analysis included *t*-tests, correlation analysis, and multiple linear regression to assess changes in FABP4 levels and their association with clinical parameters. **Results**: FABP4 and *P. gingivalis* antibody titers significantly increased with the severity of periodontitis (*p* < 0.001). After non-surgical periodontal treatment, FABP4 levels significantly decreased across all stages of periodontitis. Moderate positive correlations were observed between FABP4 and CAL, PD, GI, and *P. gingivalis* antibody titers (*p* < 0.05). Multiple linear regression showed that FABP4 levels increased significantly with the progression of periodontitis, independent of age and sex. **Conclusions**: The study indicates that FABP4 is a potential biomarker for linking periodontitis to systemic conditions such as cardiovascular diseases and diabetes. Non-surgical periodontal treatment reduced FABP4 levels, potentially contributing to the improvement of systemic conditions associated with elevated FABP4. Further research should explore the role of FABP4 in patients with periodontitis and systemic diseases to strengthen its clinical relevance.

## 1. Introduction

Noncommunicable diseases (NCDs) are a serious disease that is increasing in prevalence worldwide as the population ages and accounts for 71% of deaths worldwide each year. The comorbid presence of two or more NCDs presents a major challenge to the personal health. Periodontitis is also a NCD with a high prevalence, being the sixth most common human disease. There are some reports of an association between periodontal diseases and other NCDs. There is now a significant body of evidence to support independent associations between severe periodontitis and severe NCDs including diabetes, cardiovascular disease, chronic obstructive pulmonary disease and chronic kidney disease [1]. Therefore, rather than treating each individual disease separately, treatment can be much more effective if factors that can affect both diseases are identified because of multi-factorial interaction.

In the field of periodontal medicine, numerous studies have been undertaken to investigate the connection between periodontal diseases and systemic conditions [2]. As the American Heart Association released a position paper on the role of oral streptococci in bacterial endocoarditis in 1957, untreated periodontal disease has continued to be examined as a source of circulatory bacteria [3]. More recent study showed periodontal pathogens such as *Porphyromonas gingivalis* (*P. gingivalis*) can invade gingival tissue and access to the systemic circulation [4]. Studies have suggested that periodontal infection is a potential risk factor for cardiovascular disease (CVD), including atherosclerosis, heart attacks, and strokes [5]. Also, periodontal disease and diabetes mellitus (DM) share a bidirectional relationship, wherein diabetes mellitus acts as a risk factor for periodontal diseases, and conversely, the inflammatory mechanisms associated with periodontal diseases can negatively impact the metabolic control of diabetes [6]. Owing to the complexity of the pathogenesis or interaction of periodontal diseases and related systemic diseases, its underlying molecular mechanisms are so far unknown clearly.

Fatty aicd-binding proteins (FABPs) are a family of proteins, expressed in a tissue specific manner, that bind fatty acid ligands and are involved in shuttling fatty acids to cellular compartments, modulating intracellular lipid metabolism, amd regulating gene expression. Several members of the FABP family have been shown to have important roles in regulating metabolism and have links to the deveolpment of insulin resistance and the metabolic syndrome [7]. Fatty acid-binding protein 4 (FABP4), alternatively referred to as adipocyte FABP (A-FABP or aP2), is a 14–15 kDa protein capable of reversible binding to hydrophobic ligands, including long-chain fatty acids and various lipids [8]. FABP4 are mainly expressed in adipocytes and macrophages and regulate intracellular lipid transportation and reactions [9]. FABP4 expression was involved with Toll-like receptors (TLRs) which recognize pathogens and mediate signaling pathways important for host defense [10]. Overexpression of FABP4 promotes the secretion of inflammatory cytokines such as interleukin-1β and tumor necrosis factor-α. FABP4 is involved in the regulation of low-grade systemic and chronic inflammation, known as meta-inflammation. This condition can be associated with insulin resistance and atherosclerosis. FABPs play also an important role in carcinogenesis. Modified FABPs expression patterns were described for prostate, bladder, renal cell carcinoma and other types of cancer cells [11]. The potential of FABP4 as a therapeutic target for metabolic and cardiovascular disease has already been widely discussed [12,13,14,15]. Recently, FABP4 is being explored as a biomarker for diagnosis of various systemic diseases including prostate needle biopsy for prostate cancer, pregnancy with restricted fetal growth, and chronic obstructive pulmonary disease [16,17,18].

Periodontitis is associated with the presence of a dysbiotic microbial community in a susceptible host. Although bacteria are required in disease pathogenesis, patients are not equally susceptible and do not respond similarly to treatment. It is ultimately the host inflammatory response to the microbial challenge that primarily derives immune cell mediated self degradation of periodontal tissues resulting in eventual tooth loss [19]. Current anti-infective therapies of periodontitis that target bacterial plaque have limited success; the focus needs to shift toward host modulatory agents that promote the resolution of inflammation and the restoration of tissue homeostasis. It is necessary to identify systemic markers that respond sensitively to the severity of periodontitis to find a host modulation agent.

Based upon the current understanding of FABP and its crucial roles in inflammation, atherosclerosis and metabolism, it is hypothesized that FABP may be an alternative biomarker in clinical research investigating the association of periodontitis with CVD or DM. A pilot clinical study showed that periodontal therapy decreases serum levels of FABP4 in systemically healthy subjects [20]. Our recent laboratory study demonstrated that periodontal pathogens stimulate lipid uptake in macrophages by modulating FABP4 expression [21]. Therefore, we aim to explore the significant role of FABP4 in the severity of periodontitis and discuss its usefulness as a biomarker linking periodontitis and systemic diseases. The purpose of this study is to investigate the levels of serum FABP4 in healthy individuals without systemic disease, based on the severity of periodontitis, and to determine whether non-surgical periodontal treatment can induce changes in the levels of serum FABP4.

## 2. Materials and Methods

### 2.1. Study Design and Patient Selection

This study was performed between 2020 and 2022 at Periodontics department of Pusan National University Dental Hospital. Subjects were recruited from patients who were diagnosed with periodontitis stage I, II, or III. They were diagnosed according to 2017 classification of periodontal disease [22]. Inclusion criteria also required systemic healthy adults with 20 or more teeth. Exclusion criteria consisted of patients who had systemic diseases such as diabetes, cardiovascular disease, chronic obstructive pulmonary disease, dementia, and pregnancy. Also subjects who have received periodontal treatment within the last 6 months or patients who have taken antibiotics within the last 6 weeks were excluded. This study was approved by the Institutional Review Board (IRB) of Pusan National University Dental Hospital (IRB no. PNUDH-2020-001). The STROBE checklist was followed in preparing this manuscript.

### 2.2. Study Visit Timeline

During the first visit, a review of inclusion and exclusion criteria was conducted, and informed consent was obtained. Additionally, a review of medical history, oral examination, clinical examination, and diagnosis of periodontitis were performed. Blood sampling is conducted for every patient undergoing non-surgical periodontal treatment for FABP4 levels and *P. gingivalis* antibody titer. Clinical examinations were documented at the baseline, and scaling and root planing was performed along with oral hygiene instruction (OHI). One month after non-surgical periodontal treatment, blood sampling was conducted, and clinical examinations were documented again.

### 2.3. Blood Collection and Assay

At the baseline, 5 mL peripheral blood was sampled. The blood sample was centrifuged in the laboratory to separate the serum, and then confirmed the *P. gingivalis* antibody titer by *P. gingivalis* antibody titer ELISA kit (Boster Biological Technology, New York, NY, USA) and FABP4 levels using Human FABP4/A Fabp ELISA Kit (PicoKine^®^, Pleasanton, CA, USA). One month after the nonsurgical periodontal treatment, the aforementioned procedure was repeated in the laboratory.

### 2.4. Clinical Examination

Clinical attachment level (CAL): Distance (mm) from the cementoenamel junction of a tooth to the base of the periodontal pocket, or the sum of gingival recession and periodontal pocket depth. It explored six points (mesiobuccal, midbuccal, distobuccal, mesiolingual, midlingual, distolingual) of tooth using periodontal probe.

Probing pocket depth (PD): Distance (mm) from gingival margin to the base of perio dontal pocket. The insertion of the periodontal probe into the periodontal pocket should be parallel to the long axis of the tooth, applying 20–30 g force to ensure that the tip of the periodontal probe reaches the base of the periodontal pocket. It explored six points (mesiobuccal, midbuccal, distobuccal, mesiolingual, midlingual and distolingual) of tooth using periodontal probe.

Plaque index (PI): An Sillness & Löe plaque index indicating oral hygine. It measures only the thickness of dental plaque on the mesiobuccal, midbuccal, distobuccal and lingual side, ignoring the range of dental plaque on the tooth surface. The index is calculated based on the visible thickness of dental plaque without the use of disclosing agents.

Gingival index (GI): An Löe & Sillness gingival index for evaluating gingival inflammation. The surrounding tissues of the teeth are divided into buccal, mesiobuccal, distobuccal, and lingual aspect, and gingival inflammation in each area is observed.

### 2.5. Statistical Analysis

Statistical analysis of the clinical data was performed by jamovi (version 2.3.28). The ideal sample size was calculated considering the change in serum levels of FABP4, with a significant difference of 2.0 ng/mL between groups, a standard deviation of 1.5 ng/mL, a 5% significance level (α = 0.05), and 95% power. A total of 15 subjects in each group were required. Baseline demographic and clinical characteristics were calculated according to periodontal stage using means with standard error (mean ± SE). Differences in FABP4 and *P. gingivalis* antibody titer at baseline and post-treatment within each periodontal stage, as well as differences in clinical parameters at baseline and post-treatment, were estimated using *t*-tests for continuous variables at a significance level of α = 0.05. Correlation analysis was performed between clinical parameters and FABP4 as well as *P. gingivalis* antibody titer and FABP4. The association of risk variables with FABP4 was assessed using multiple linear regression analysis.

## 3. Results

**Figure 1:**
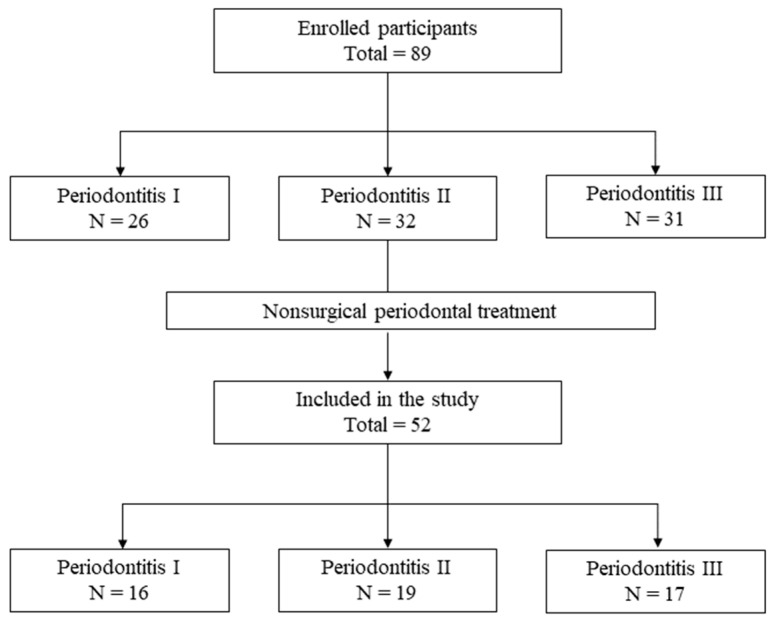
Flowchart showing the experimmental design.

Eighty-nine individuals diagnosed with periodontitis agreed to take part in the research at the baseline. After periodontal treatment, in each stage, ten, thirteen, and fourteen subjects respectively either did not meet the inclusion criteria or chose not to participate. A total of fifty-two subjects successfully underwent the non-surgical treatment protocol without encountering any significant adverse events. Among these participants, 16 were classified as periodontitis stage I, 19 as periodontitis stage II, and 17 as periodontitis stage III, following the 2017 classification of periodontal disease (Figure 1).

At the baseline, there was a notable significance in the CAL and PD difference in each periodontal group (*p* < 0.001), whereas the PI and GI did not show statistical significance. FABP4 and *P. gingivalis* antibody titer level was different in each stage significantly (*p* < 0.001). As the severity of periodontitis increased, serum FABP4 and *P. gingivalis* antibody titer level increased (Table 1).

In all patients with periodontitis, all post-treatment indicators showed statistically significant improvement compared to before treatment (Table 2A).

**Figure 2:**
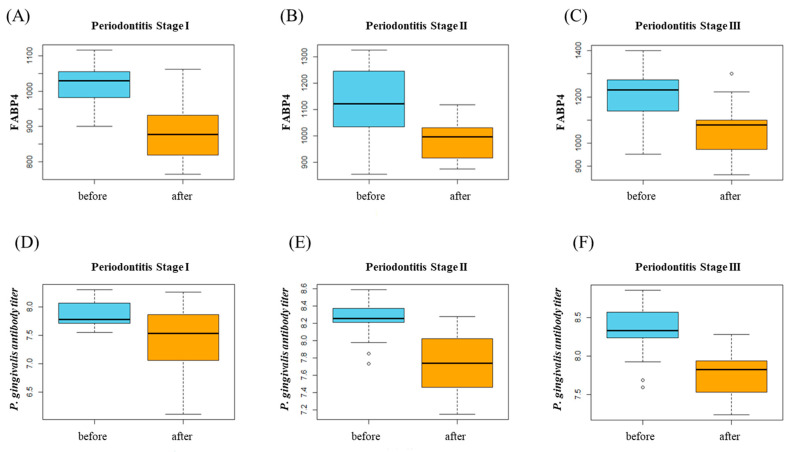
(**A**–**C**) FABP4 changes after periodontal treatment in each stage. (**D**–**F**) *P. gingivalis* antibody titer level changes after periodontal treatment in each stage.

When comparing clinical parameters after the periodontal treatment in each stage, several parameters were statistically significant. On the other hand, FABP4 and *P. gingivalis* antibody titier level change was significant in all group (Table 2B, Figure 2).

**Figure 3:**
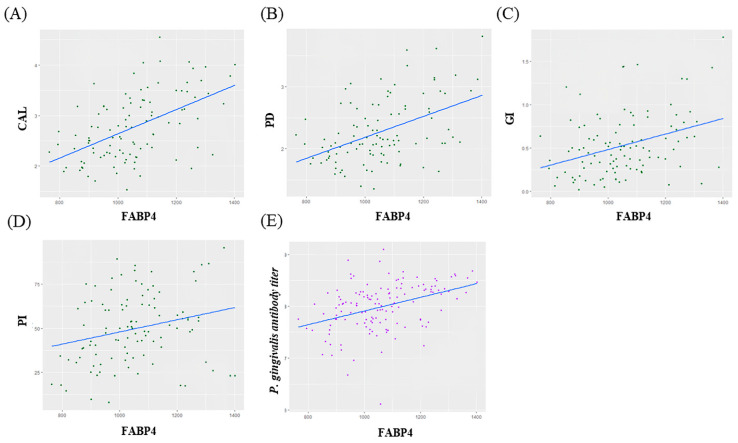
(**A**–**D**) Correlations between changes in FABP4 and Clinical parameters. (**E**) Correlations between changes in FABP4 and *P. gingivalis* antibody titer.

Correlation analysis showed that there was moderate positive correlation between FABP4 and CAL, PD, GI and *P. gingivalis* antibody titier level (CAL; ρ = 0.52, *p* < 0.05, PD; ρ = 0.47, *p* < 0.05, GI; ρ = 0.35, *p* < 0.05, *P. gingivalis* antibody titer; ρ = 0.39, *p* < 0.05) (Figure 3).

The multiple linear regression model to determine the risk variables with FABP4 was done (Table 3). The explanatory power of the fitted regression model for the variation in FABP4 is 35.8%. The regression coefficients of age and sex are not statistically significant. After controlling for age and sex, the estimated FABP4 level in stage II is 132.93 higher than in stage I, while stage III shows an increase of 81.83 compared to stage II. The *p*-values for these differences are 0.001 and 0.033, respectively, indicating statistical significance.

## 4. Discussion

Periodontal diseases are the result of a process initiated by a local dysbiotic microbial community, which triggers a host immune response that is influenced by environmental and genetic factors as well as the virulence of the local microbiome. Although they are initially caused by microbial biofilm, environmental and genetic factors contribute to their development. The important role of the local host immune response in the pathogenesis of periodontitis was revealed [23]. Periodontal tissue destruction begins with an inflammatory process caused by oral bacterial infection. Host susceptibility is a decisive factor in the development and progression of periodontitis. Pathogens induce periodontitis in susceptible patients and most of the time, the immune system is very efficient and prevents disease progression until the microbial dysbiotic environment has been established. Recently, much attention has been drawn to regulating the immune response to putative periodontal pathogens in order to resolve inflammation, control the osteolytic environment, and restore physiologic bone formation. Therefore, in diagnosing and treating periodontitis, identifying and modulating the host’s inflammatory and immune responses could be a promising strategy [24].

Periodontal infection increases the circulation of virulence factors from periodontal pathogens, such as lipopolysaccharide, which act as systemic inflammation and contribute to metabolic disturbances. Lipopolysaccharide in the blood stream causes endotoxemia that is associated with low-grade inflammation [25]. Persistent low-grade inflammation is associated with chronic cardiometabolic disorders, such as obesity, nonalcoholic fatty liver disease, metabolic syndrome, insulin resistance, type 2 diabetes, dyslipidemia, and cardiovascular disease [25,26,27,28]. Research has demonstrated that lipopolysaccharide notably enhances the synthesis of FABP4 by macrophages via the toll-like receptor pathways [10]. Also, macrophages infected by *P. gingivalis* accumulate fatty acids, leading to an increase in FABP4 levels, which may initiate and/or contribute to the progression of systemic diseases like foam cell formation in atherosclerosis [9,29,30]. Increased FABP4 level independently predicts atherosclerosis, evaluated by carotid intima-media thickness, a recognized marker of the disease [29]. These studies are part of the process of finding the mechanisms that influence the relationship between periodontitis and systemic diseases.

Clinical parameters such as CAL, PD, GI as well as *P. gingivalis* antibody titer has moderate positive correlation with serum FABP4. In a multiple linear regression analysis while controlling other variables (i.e., age, sex), there is a statistically significant increase in FABP4 levels as the periodontal stage increases. These pieces of evidence clinically indicate that periodontitis is related to serum FABP4. Therefore, we consider the potential use of FABP4 as a biomarker for diagnosis and predicting stage of periodontitis.

A recent study revealed a significant association between FABP4 expression and c-Jun N-terminal kinase (JNK) activation [31]. Although the classical understanding of fatty acids is preserving basic cell structure and supporting energy metabolism, current research indicates their involvement in cell signaling cascades [32]. Targets that may be influenced by lipids and are associated with inflammation and insulin signaling include stress-activated kinases like JNK and inhibitor of nuclear kappa B kinase-nuclear factor-kappa B (IKK/NFκB), as well as nuclear hormone receptors such as peroxisome proliferator-activated receptor (PPARγ) and liver X receptor α (LXRα) [32]. Among these, activation of JNK influenced the mRNA and protein expression of FABP4 in macrophages infected with periodontal pathogens [31].

FABP4, which is also found in adipocytes, regulates lipid transport and metabolism, which is considered to link obesity to various systemic diseases [21]. Serum FABP4 levels are negatively correlated with glucose disposal rate (GDR), a marker of insulin resistance in skeletal muscle, as well as with serum insulin levels. In individuals with type 2 diabetes, FABP4 concentration was higher compared to those without diabetes, a condition commonly associated with endothelial dysfunction [33]. When FABP4 levels decrease in diabetic patients, the reactive hyperemia index (RHI), which is a marker for evaluating endothelial function, increases [33]. In our study, we confirmed that FABP4 levels were decreased after non-surgical periodontal treatment, suggesting that periodontal treatment may affect various systemic functions associated with FABP4 as mentioned above.

Targeting the secreted FABP4 may represent an effective therapeutic strategy against metabolic, cardiovascular and other diseases. FABP4 silencing reduces LPS-induced cardiomyocyte hypertrophy and apoptosis by down-regulating the TLRs [34]. Small molecule inhibitors of FABP4 have attracted interest following the recent publications of beneficial pharmacological effects of these compounds. Hundreds FABP4 inhibitors have been synthesized for effective atherosclerosis and diabetes treatments, including derivatives of niacin, quinoxaline, aryl-quinoline, bicyclic pyridine, urea, aromatic and other novel heterocyclic compounds [35]. Small molecules that inhibit FABP4 mediated responses, might serve as potential candidates for the treatment of different components of metabolic syndromes, such as insulin resistance, type 2 diabetes, and atherosclerosis. Nonsurgical periodontal therapy with or without antimicrobials mechanically removes etiologic dental biofilm and remains gold standard treatment; however, targeting only microbes does not achieve favorable outcomes in all periodontal patients. The adjunctive use of host modulatory agents can have a positive impact on the progression of periodontal disease, especially in susceptible patients who develop a chronic inflammatory response against the microbiome associated with genetic, systemic, or environmental factors and for whom conventional therapeutic approached are not effective. Periodontists have a desire to control the host’s inflammation and immune response through host modulation therapy along with mechanical treatment in treating periodontal disease. This is especially important for patients with periodontal disease who also have systemic diseases such as NCDs. However, there is no promising agent widely available for host modulating yet. We suggest that molecules that inhibit FABP4, which is associated with periodontitis and systemic inflammation, may be helpful in the treatment of periodontitis.

Biomarkers can be powerful tools in disease management. Many clinicians are trying to find effective biomarkers for specific diseases. Clinical biomarker for the diagnosis of periodontitis have not yet been established. Diagnosis of periodontitis is still dependent on combinations of clinical examination including PD, CAL, GI and radiographs which are prone to discrepancies. This often depends on several factors including operator’s skill, direction and angle of measurement, applied force, and dimension/shape of the probe. One of the updates to the new classification system for periodontal disease was the addition of a periodontitis grade to help determine the rate of progression and predict prognosis. This domain of the diagnostic statement is calculated by using patient’s age and percent of periodontal bone loss at the worst site [22]. In managing periodontal disease, it is necessary to use multiple biomarkers or find other biomarkers that are stable and have low vulnerability. There have been numerous studies exploring the use of various salivary and gingival crevicular fluid biomarkers to diagnose periodontitis. Biomarkers such as cytokines, matrix metalloproteinases (MMPs), and C-reactive protein (CRP) have been reported to reflect the inflammatory state associated with periodontitis [36,37,38,39]. However, these salivary biomarkers are subject to variability due to several individual factors, including oral hygiene status and saliva composition [40,41]. Additionally, some biomarkers may be present at low concentrations, which can limit their diagnostic utility [42]. In contrast, blood biomarkers are likely to be present at higher concentrations compared to salivary biomarkers, allowing for more accurate and sensitive detection. In this study, differences in the concentrations of metabolic factors were observed even in stages of periodontitis with low severity Additionally, blood biomarkers offer the advantage of being able to monitor disease progression and response to treatment. Therefore, utilizing blood biomarkers has the potential to significantly enhance diagnostic accuracy and treatment monitoring for periodontal diseases.

There are few studies suggesting the use of FABP4 as a biomarker related to periodontitis. To the best of our knowledge, X. Li et al. [19] was the only previous investigation into the association between FABP4 and periodontitis. The previous study examined whether the treatment of periodontitis affects the level of FABP4 within the limitation of small sample size and irrespective of the severity of periodontitis. We propose FABP4 as a potential biomarker for the evaluation of periodontitis. Our study aimed to investigate the relationship between FABP4 and *P. gingivalis* antibody titer according to the stage of periodontitis. In our study, FABP4 and *P. gingivalis* antibody titer decreased after treatment across all stages. As periodontal treatment results in a decrease in FABP4, it implies that several systemic conditions mentioned earlier may improve. Our study results showed that as periodontitis became more severe, serum FABP4 levels, is involved in the regulation of low-grade systemic and chronic inflammation, increased. Therefore, we can cautiously infer that periodontal infection may trigger low grade systemic inflammation. The decrease in serum FABP4 after non-surgical periodontal treatment suggested that low grade systemic inflammation could be managed considerably well by non-surgical periodontal treatment.

In this study, we measured FABP4 levels in periodontitis patients without systemic diseases to excluding various confounding factors of systemic diseases. It also includes the hypothesis that patients with periodontitis who did not have systemic diseases may have elevated FABP4 levels due to periodontitis, which may be a risk factor for the development of systemic diseases. Since FABP4 can be considered as a remarkable biomarker in patients with diabetes or atherosclerosis, it would be more meaningful to select periodontitis patients with systemic diseases to explore the linking biomarker of periodontitis and systemic diseases. A better understanding of the FABP4 will improve preventive, diagnostic, therapeutic and reparative strategies for periodontitis and related systemic disease. Future studies will investigate the changes in FABP4 markers and periodontal markers in patients with diabetes or atherosclerosis who also have periodontitis.

## 5. Conclusions

Within the limitation of our study, the host serum FABP4 levels increased with increasing periodontitis severity. Non-surgical periodontal treatment led to decrease in serum FABP4 level across all stages of periodontitis. Decreasing level of serum FABP4 could make improvements in several systemic conditions associated with elevated FABP4. We have taken a step closer to discovering the mechanism by which periodontal inflammation can affect systemic diseases such as cardiovascular diseases and diabetes.

## Figures and Tables

**Table 1:** Baseline demographic and clinical characteristics according to periodontal stage.

Variables	Stage I (n = 26)	Stage II (n = 32)	Stage III (n = 31)	*p*-Value
Age (years)	50.0 ± 2.40	58.7 ± 1.62	52.9 ± 1.73	0.007
Sex				
Female	13	21	11	
Male	13	11	20	
BMI (${kg/m}^{2}$)	23.09 ± 0.62	23.03 ± 0.52	24.26 ± 0.59	0.25
Clinical attachment level (mm)	2.40 ± 0.070	2.91 ± 0.110	3.63 ± 0.189	<0.001
Probing pocket depth (mm)	2.19 ± 0.076	2.37 ± 0.101	2.87 ± 0.108	<0.001
Plaque index	0.563 ± 0.038	0.543 ± 0.035	0.561 ± 0.047	0.912
Gingival index	0.510 ± 0.072	0.641 ± 0.076	0.849 ± 0.110	0.043
FABP4 (pg/mL)	1002 ± 12.8	1113 ± 19.5	1191 ± 23.0	<0.001
*P. gingivalis* antibody titer (Mean ${log}_{10}\;$antibody titers of *P. gingivalis*)	7.90 ± 0.046	8.27 ± 0.047	8.36 ± 0.058	<0.001

**Table 2:** (A) Comparison of indicators before and after non-surgical periodontal treatment. (B) Comparison of indicators before and after periodontal treatment, catagorized by stage.

(A)									
	Before			After			*p*-Value		
Clinical attachment level (mm)	2.96			2.57			<0.001		
Probing pocket depth (mm)	2.45			2.08			<0.001		
Plaque index	55.81			43.65			<0.001		
Gingival index	0.691			0.369			<0.001		
FABP4 (pg/mL)	1120.983			982.134			<0.001		
*P. gingivalis* antibody titer (Mean ${log}_{10}\;$antibody titers of *P. gingivalis*)	8.167			7.647			<0.001		
**(B)**									
	**Stage I**			**Stage II**			**Stage III**		
	**Before**	**After**	***p*** **-Value**	**Before**	**After**	***p*-Value**	**Before**	**After**	***p*-Value**
Clinical attachment level (mm)	2.348	2.145	0.095	2.942	2.596	0.001	3.544	2.947	<0.001
Probing pocket depth (mm)	2.123	1.986	0.246	2.296	1.986	0.002	2.974	2.272	<0.001
Plaque index	57.908	37.066	0.003	54.348	48.590	0.274	55.457	44.316	0.082
Gingival index	0.574	0.134	0.002	0.631	0.343	0.001	0.869	0.449	0.002
FABP4 (pg/mL)	1016.056	887.306	<0.001	1127.308	1121.444	<0.001	1212.668	1066.771	<0.001
*P. gingivalis* antibody titer (Mean ${log}_{10}\;$antibody titers of *P. gingivalis*)	7.879	7.393	0.006	8.249	7.741	<0.001	8.329	7.764	<0.001

**Table 3:** Result of Multiple Linear Regression Analysis of risk variables with FABP4.

Independent Variable	Β (95% CI)	S.E.	*t*-Value	*p*-Value	Adjusted R^2^
Model					0.358 (*p* < 0.001)
Age	−2.84	1.51	−1.54	0.130	
Sex					
M–F	−44.00	31.89	−1.38	0.174	
Stage					
I–II	−132.93	37.88	−3.51	0.001	
III–II	81.83	37.28	2.20	0.033	

## Data Availability

The original contributions presented in this study are included in the article. Further inquiries can be directed to the corresponding author.

## References

[1] Sanz M., Marco Del Castillo A., Jepsen S., Gonzalez-Juanatey J.R., D’Aiuto F., Bouchard P., Chapple I., Dietrich T., Gotsman I., Graziani F. (2020). Periodontitis and cardiovascular diseases: Consensus report. J. Clin. Periodontol..

[2] Williams R.C., Offenbacher S. (2000). Periodontal medicine: The emergence of a new branch of periodontology. Periodontol. 2000.

[3] Kumar P.S. (2017). From focal sepsis to periodontal medicine: A century of exploring the role of the oral microbiome in systemic disease. J. Physiol..

[4] Zhang Z., Liu D., Liu S., Zhang S., Pan Y. (2020). The Role of *Porphyromonas gingivalis* Outer Membrane Vesicles in Periodontal Disease and Related Systemic Diseases. Front. Cell Infect. Microbiol..

[5] Tonetti M.S. (2009). Periodontitis and risk for atherosclerosis: An update on intervention trials. J. Clin. Periodontol..

[6] Falcao A., Bullon P. (2019). A review of the influence of periodontal treatment in systemic diseases. Periodontol. 2000.

[7] Boord J.B., Fazio S., Linton M. (2002). Cytoplasmic fatty acid-binding proteins: Emerging roles in metabolism and atherosclerosis. Curr. Opin. Lipidol..

[8] Furuhashi M., Hotamisligil G.S. (2008). Fatty acid-binding proteins: Role in metabolic diseases and potential as drug targets. Nat. Rev. Drug Discov..

[9] Hotamisligil G.S. (2017). Inflammation, metaflammation and immunometabolic disorders. Nature.

[10] Kazemi M.R., McDonald C.M., Shigenaga J.K., Grunfeld C., Feingold K.R. (2005). Adipocyte fatty acid-binding protein expression and lipid accumulation are increased during activation of murine macrophages by toll-like receptor agonists. Arterioscler. Thromb. Vasc. Biol..

[11] Nieman K.M., Kenny H.A., Penicka C.V., Ladanyi A., Buell-Gutbrod R., Zillhardt M.R., Romero I.L., Carey M.S., Mills G.B., Hotamisligil G.S. (2022). Adipocytes promote ovarian cancer metastasis and provide energy for rapid tumor growth. Nat. Med..

[12] Furuhashi M. (2019). Fatty Acid-Binding Protein 4 in Cardiovascular and Metabolic Diseases. J. Atheroscler. Thromb..

[13] Furuhashi M., Ishimura S., Ota H., Miura T. (2011). Lipid chaperones and metabolic inflammation. Int. J. Inflam..

[14] Furuhashi M., Saitoh S., Shimamoto K., Miura T. (2014). Fatty Acid-Binding Protein 4 (FABP4): Pathophysiological Insights and Potent Clinical Biomarker of Metabolic and Cardiovascular Diseases. Clin. Med. Insights Cardiol..

[15] Hotamisligil G.S., Bernlohr D.A. (2015). Metabolic functions of FABPs--mechanisms and therapeutic implications. Nat. Rev. Endocrinol..

[16] Harraz A.M., Atia N., Ismail A., Shady A., Farg H., Gabr H., Fouda M., Abol-Enein H., Abdel-Aziz A.F. (2020). Evaluation of serum fatty acid binding protein-4 (FABP4) as a novel biomarker to predict biopsy outcomes in prostate biopsy naïve patients. Int. Urol. Nephrol..

[17] Bolluk G., Oğlak S.C., Kayaoğlu Yıldırım Z., Zengi O. (2024). Maternal serum fatty acid binding protein-4 level is upregulated in fetal growth restriction with abnormal doppler flow patterns. J. Obstet. Gynaecol. Res..

[18] Aslani M.R., Ghazaei Z., Ghobadi H. (2020). Correlation of serum fatty acid binding protein-4 and interleukin-6 with airflow limitation and quality of life in stable and acute exacerbation of COPD. Turk. J. Med. Sci..

[19] Van Dyke T.E. (2020). Shifting the paradigm from inhibitors of inflammation to resolvers of inflammation in periodontitis. J. Periodontol..

[20] Li X., Tse H.F., Yiu K.H., Zhang C., Jin L.J. (2013). Periodontal therapy decreases serum levels of adipocyte fatty acid-binding protein in systemically healthy subjects: A pilot clinical trial. J. Periodontal Res..

[21] Kim D.J., Rho J.H., Woo B.H., Joo J.Y., Lee J.Y., Song J.M., Lee J.H., Park H.R. (2019). Periodontal Pathogens Modulate Lipid Flux via Fatty Acid Binding Protein 4. J. Dent. Res..

[22] Tonetti M.S., Greenwell H., Kornman K.S. (2018). Staging and grading of periodontitis: Framework and proposal of a new classification and case definition. J. Periodontol..

[23] Teles F., Collman R.G., Mominkhan D., Wang Y. (2022). Viruses, periodontitis, and comorbidities. Periodontol. 2000.

[24] Yang B., Pang X., Li Z., Chen Z., Wang Y. (2021). Immunomodulation in the treatment of periodontitis: Progress and perspectives. Front. Immunol..

[25] Pussinen P.J., Kopra E., Pietiäinen M., Lehto M., Zaric S., Paju S., Salminen A. (2022). Periodontitis and cardiometabolic disorders: The role of lipopolysaccharide and endotoxemia. Periodontol. 2000.

[26] Pussinen P.J., Tuomisto K., Jousilahti P., Havulinna A.S., Sundvall J., Salomaa V. (2007). Endotoxemia, immune response to periodontal pathogens, and systemic inflammation associate with incident cardiovascular disease events. Arterioscler. Thromb. Vasc. Biol..

[27] Tomas I., Diz P., Tobias A., Scully C., Donos N. (2012). Periodontal health status and bacteraemia from daily oral activities: Systematic review/meta-analysis. J. Clin. Periodontol..

[28] Forner L., Larsen T., Kilian M., Holmstrup P. (2006). Incidence of bacteremia after chewing, tooth brushing and scaling in individuals with periodontal inflammation. J. Clin. Periodontol..

[29] Furuhashi M., Yuda S., Muranaka A., Kawamukai M., Matsumoto M., Tanaka M., Moniwa N., Ohnishi H., Saitoh S., Shimamoto K. (2018). Circulating Fatty Acid-Binding Protein 4 Concentration Predicts the Progression of Carotid Atherosclerosis in a General Population Without Medication. Circ. J..

[30] Furuhashi M., Fucho R., Gorgun C.Z., Tuncman G., Cao H., Hotamisligil G.S. (2008). Adipocyte/macrophage fatty acid-binding proteins contribute to metabolic deterioration through actions in both macrophages and adipocytes in mice. J. Clin. Investig..

[31] Makowski L., Hotamisligil G.S. (2004). Fatty acid binding proteins--the evolutionary crossroads of inflammatory and metabolic responses. J. Nutr..

[32] Nakamura R., Okura T., Fujioka Y., Sumi K., Matsuzawa K., Izawa S., Ueta E., Kato M., Taniguchi S.I., Yamamoto K. (2017). Serum fatty acid-binding protein 4 (FABP4) concentration is associated with insulin resistance in peripheral tissues, A clinical study. PLoS ONE.

[33] Aragonès G., Ferré R., Lázaro I., Cabré A., Plana N., Merino J., Heras M., Girona J., Masana L. (2010). Fatty acid-binding protein 4 is associated with endothelial dysfunction in patients with type 2 diabetes. Atherosclerosis.

[34] Sun F., Chen G., Yang Y., Lei M. (2021). Fatty acid-binding protein 4 silencing protects against lipopolysaccharide-induced cardiomyocyte hypertrophy and apoptosis by inhibiting the Toll-like receptor 4-nuclear factor-кB pathway. Int. J. Med. Res..

[35] Floresta G., Pistarà V., Amata E., Dichiara M., Marrazzo A., Prezzavento O., Rescifina A. (2017). Adipocyte fatty acid binding protein 4 (FABP4) inhibitors. A comprehensive systematic review. Eur. J. Med. Chem..

[36] Zhang L., Henson B.S., Camargo P.M., Wong D.T. (2009). The clinical value of salivary biomarkers for periodontal disease. Periodontol. 2000.

[37] Morelli T., Stella M., Barros S.P., Marchesan J.T., Moss K.L., Kim S.J., Yu N., Aspiras M.B., Ward M., Offenbacher S. (2014). Salivary biomarkers in a biofilm overgrowth model. J. Periodontol..

[38] Pederson E.D., Stanke S.R., Whitener S.J., Sebastiani P.T., Lamberts B.L., Turner D.W. (1995). Salivary levels of alpha 2-macroglobulin, alpha 1-antitrypsin, C-reactive protein, cathepsin G and elastase in humans with or without destructive periodontal disease. Arch. Oral. Biol..

[39] Herr A.E., Hatch A.V., Throckmorton D.J., Tran H.M., Brennan J.S., Giannobile W.V., Singh A.K. (2007). Microfluidic immunoassays as rapid saliva-based clinical diagnostics. Proc. Natl. Acad. Sci. USA.

[40] Zhang Y., Kang N., Xue F., Qiao J., Duan J., Chen F., Cai Y. (2021). Evaluation of salivary biomarkers for the diagnosis of periodontitis. BMC Oral. Health.

[41] Lee J., Lee J.-B., Song H.-Y., Son M.J., Li L., Rhyu I.-C., Lee Y.-M., Koo K.-T., An J.-S., Kim J.S. (2020). Diagnostic Models for Screening of Periodontitis with Inflammatory Mediators and Microbial Profiles in Saliva. Diagnostics.

[42] Teles F.R.F., Chandrasekaran G., Martin L., Patel M., Kallan M.J., Furquim C., Hamza T., Cucchiara A.J., Kantarci A., Urquhart O. (2024). Salivary and serum inflammatory biomarkers during periodontitis progression and after treatment. J. Clin. Periodontol..

